# Long-Term Low-Dose Delta-9-Tetrahydrocannbinol (THC) Administration to Simian Immunodeficiency Virus (SIV) Infected Rhesus Macaques Stimulates the Release of Bioactive Blood Extracellular Vesicles (EVs) that Induce Divergent Structural Adaptations and Signaling Cues

**DOI:** 10.3390/cells9102243

**Published:** 2020-10-06

**Authors:** Yuan Lyu, Steven Kopcho, Mahesh Mohan, Chioma M. Okeoma

**Affiliations:** 1 Department of Pharmacology, Stony Brook University Renaissance School of Medicine, Stony Brook, NY 11794-8651, USA; yuan.lyu@stonybrook.edu (Y.L.); steven.kopcho@stonybrook.edu (S.K.); 2 Host Pathogen Interaction, Southwest National Primate Research Center, Texas Biomedical Research Institute, San Antonio, TX 78227-5302, USA

**Keywords:** delta-9-tetrahydrocannbinol (THC), SIV/HIV, extracellular vesicles (EVs)

## Abstract

Blood extracellular vesicles (BEVs) carry bioactive cargo (proteins, genetic materials, lipids, licit, and illicit drugs) that regulate diverse functions in target cells. The cannabinoid drug delta-9-tetrahydrocannabinol (THC) is FDA approved for the treatment of anorexia and weight loss in people living with HIV. However, the effect of THC on BEV characteristics in the setting of HIV/SIV infection needs to be determined. Here, we used the SIV-infected rhesus macaque model of AIDS to evaluate the longitudinal effects of THC (THC/SIV) or vehicle (VEH/SIV) treatment in HIV/SIV infection on the properties of BEVs. While BEV concentrations increased longitudinally (pre-SIV (0), 30, and 150 days post-SIV infection (DPI)) in VEH/SIV macaques, the opposite trend was observed with THC/SIV macaques. SIV infection altered BEV membrane properties and cargo composition late in infection, since i) the electrostatic surface properties (zeta potential, ζ potential) showed that RM BEVs carried negative surface charge, but at 150 DPI, SIV infection significantly changed BEV ζ potential; ii) BEVs from the VEH/SIV group altered tetraspanin CD9 and CD81 levels compared to the THC/SIV group. Furthermore, VEH/SIV and THC/SIV BEVs mediated divergent changes in monocyte gene expression, morphometrics, signaling, and function. These include altered tetraspanin and integrin β1 expression; altered levels and distribution of polymerized actin, FAK/pY397 FAK, pERK1/2, cleaved caspase 3, proapoptotic Bid and truncated tBid; and altered adhesion of monocytes to collagen I. These data indicate that HIV/SIV infection and THC treatment result in the release of bioactive BEVs with potential to induce distinct structural adaptations and signaling cues to instruct divergent cellular responses to infection.

## 1. Introduction

Cannabinoids, the chemical compounds found in the cannabis (marijuana) plant, exhibit pharmacobiological effects in various conditions, including remyelination [1], hyperthermia [2], hypothermia [3], catalepsy [4,5], inflammation and immune activation [6,7,8,9,10,11,12], analgesia [13], cellular growth/arrest, apoptosis [14], as well as alteration in the functions of a variety of immune cells [15,16,17,18]. Although more than 100 cannabinoids have been identified in cannabis, THC and the non-psychotropic cannabidiol (CBD) are the most widely used and both have the same molecular formula (C_21_H_30_O_2_) and molecular weight (314.5 g/mol). The therapeutic actions of THC are based on its antiemetic, analgesic, and anti-inflammatory activities. 

Cannabinoid use is frequent in HIV-infected individuals, both as a recreational agent and an appetite stimulant [19,20,21,22,23]. Controlled studies in SIV-infected RMs showed that chronic cannabinoid treatment slowed disease progression, prolonged survival, and attenuated infection-induced inflammation [24,25]. Moreover, high-intensity cannabis-smoking HIV-infected individuals had reduced plasma HIV viral load [26], circulating CD16^+^ monocytes, and plasma IP-10 levels [27], observations that confirmed the findings in SIV-infected RMs [24]. Indeed, the protective effects of chronic THC administration in the RM intestine involve selective modulation of anti-inflammatory miRNA expression [8]. 

There are two known cannabinoid receptors—cannabinoid receptor type 1 (CB1R, CB1) that localizes primarily in the central nervous system (CNS) and the testis, and cannabinoid receptor type 2 (CB2R, CB2) that localizes primarily in hematopoietic cells and moderately in specific brain areas and peripheral cells. Activation of CB1 is responsible for the psychotropic effects of cannabinoids, while CB2 activation is involved in its anti-inflammatory and immunomodulatory properties. Interestingly, activation of CB2 orchestrates molecular crosstalk similar to those triggered by CB1 activation [28,29]. Activation of CB2 ameliorates neurocognitive impairments and decreases neuronal damage associated with HIV infection through inhibition of viral replication and suppression of the activity of neurotoxic HIV Tat and HIV-1 gp120 [30,31]. Moreover, CB2 activation inhibits inflammation, barrier permeability, leukocyte infiltration, and Tat-mediated monocyte migration through the hematoencephalic barrier [31]. Although both THC and CBD have anti-inflammatory properties, CBD does not exert psychotropic effects and owing to its negative allosteric effects on CB1 [32] may be beneficial when combined with THC to reduce its psychotropic and enhance its therapeutic effects.

Being a CB1 and CB2 partial agonist with an agonist–antagonist activity [33], the impact of THC on HIV/SIV pathogenesis is a promising research topic, which continues to evolve. Further, there is a significant knowledge gap in the contribution of extracellular vesicles (EVs) to the effects of THC in the setting of HIV/SIV infection. EVs are a key player in the pathogenesis of viral infections [34,35,36,37,38,39,40,41,42,43,44]. EVs are cargo-carrying, quasi-nanovesicles that mediate distal and proximal intercellular communications [45,46,47]. EVs are released by many cell types [48], and are present in all body fluids [36,45,46,47,48,49,50,51,52,53,54,55,56]. EVs also carry markers of the producer cells. As a result, depending on whether the producer cells are healthy or pathologic, EVs will carry markers corresponding to the state of the cells [40,43]. With respect to drug use, EVs have been used to establish signatures linked to methamphetamine, heroin, and alcohol abuse [57,58,59]. EVs released by substance-using HIV-infected individuals are known to exhibit pathogenic properties, including increases in cell adhesion, actin reorganization, secretion of metalloproteases, and chemotactic migration toward the HIV secretome [42]. Thus, it is likely that EVs orchestrate communication between various organs. It is also reasonable to assume that cannabinoid signaling is present in cells that produce EVs and that cannabinoids may mediate their effects via EVs since all eukaryotic cells release EVs [60,61]. It has previously been shown that EVs were detected in the blood of RMs and humans infected with SIV [62] or HIV [34,38]. However, the longitudinal effect of HIV/SIV infection on EV release, cargo, and function is lacking. Also lacking is the effect of THC on EV properties. In the present study, we used the SIV-infected rhesus macaque model to longitudinally (pre-SIV, 30, and 150 DPI) evaluate the effects of SIV and THC on BEV properties. While there are different types of EVs, with exosomes and microvesicles being the most abundant, these EVs are similar in size, composition, and functions and are difficult to distinguish and isolate in preparative quantities. Given their great similarities, we will use the term EVs in this study. 

## 2. Materials and Methods

### 2.1. Animal Care, Ethics and Experimental Procedures 

All experiments using rhesus macaques were approved by the Tulane Institutional Animal Care and Use Committee (Protocol No-3581 and 3781). The Tulane National Primate Research Center (TNPRC) is an Association for Assessment and Accreditation of Laboratory Animal Care International accredited facility (AAALAC #000594). The NIH Office of Laboratory Animal Welfare assurance number for the TNPRC is A3071-01. All clinical procedures, including administration of anesthesia and analgesics, were carried out under the direction of a laboratory animal veterinarian. Animals were anesthetized with ketamine hydrochloride for blood collection procedures. Animals were pre-anesthetized with ketamine hydrochloride, acepromazine, and glycopyrrolate, intubated and maintained on a mixture of isoflurane and oxygen. All possible measures were taken to minimize the discomfort of all the animals used in this study. Tulane University complies with NIH policy on animal welfare, the Animal Welfare Act, and all other applicable federal, state and local laws. Stony Brook University approved the use of RM specimens. All experiments were performed in accordance with the approved institutional guidelines and regulations.

### 2.2. Blood Sample Collection

Nineteen age- and weight-matched male Indian rhesus macaques (Table 1) were randomly distributed into three groups. Group 1 (*n* = 7) received twice-daily injections of vehicle (VEH) (1:1:18 of emulphor: alcohol: saline) and were infected intravenously with 100 times the 50% tissue culture infective dose (100TCID_50_) of SIVmac251. Group 2 (*n* = 7) received twice-daily injections of Δ^9^-THC for four weeks prior to SIV infection. Group 3 (*n* = 3) received twice-daily injections of Δ^9^-THC initiated at the same time as groups 1 and 2 but remained uninfected. Chronic administration of Δ^9^-THC or VEH was initiated four weeks before SIV infection at 0.18 mg/kg, as described in previous studies [26,27]. This dose of Δ^9^-THC was found to eliminate responding in a complex operant behavioral task in almost all animals [27]. The dose was subsequently increased for each subject to 0.32 mg/kg over a period of approximately two weeks when responding was no longer affected by 0.18 mg/kg on a daily basis (i.e., tolerance developed) and maintained for the duration of the study. The optimization of the THC dosing in rhesus macaques accounts for the development of tolerance during the initial period of administration. Because this dose of THC showed protection in our previously published studies [10], the same dose was used in this study. SIV levels in plasma were quantified by using the TaqMan One-Step Real-Time RT-qPCR assay that targeted the long terminal repeats (LTRs) gene [10]. Blood samples were collected monthly in 4.9 mL EDTA containing vacutainer tubes following the standard operating protocols established for blood collection from non-human primates at the TNPRC. Blood tubes were centrifuged at 2000 rpm for 10 min to separate plasma for EV studies. 

### 2.3. Cells and Chemical Reagents

Human U937 monocyte-like cells were obtained from American Type Culture Collection (ATCC) and maintained in complete RPMI media. The complete media was supplemented with 10% exosome-depleted FBS, 1% penicillin-streptomycin, 1 µg/mL amphotericin B, 2 mM sodium pyruvate, 1% glutamate and 10 mM 4-(2-hydroxyethyl)-1-piperazineethanesulfonic acid (HEPES) buffer at pH 8. The chemical reagents, provider name and contact, and instrument used are shown in Table 2.

### 2.4. Isolation of Blood Extracellular Vesicles (BEVs)

The EV isolation method has been described previously [42]. Briefly, 51 blood plasma samples from three groups were thawed at room temperature for 30 min before further process. Blood plasma were first clarified through centrifugation at 2000× *g* for 10 min followed by 10,000× *g* for 30 min to remove cellular debris and large vesicles. BEVs were purified by size-exclusion chromatography (SEC). A volume of 200 µL clarified plasma was loaded onto Sephadex G-50 fine beads packed in a 22 cm × 1 cm Econo-column and eluted by gravity using 1× DPBS. BEV fractions were collected based on the absorbance profile at 280 and 600 nm, where the first peak corresponds to BEVs. Collected BEVs were stored at −80 °C until further experiment. The isolated vesicles are called BEVs to accommodate both exosomes and microvesicles, since the vesicles are closely related in size and cargo composition. 

### 2.5. Nanoparticle Tracking Analysis (NTA) 

BEV size, concentration and ζ potential were measured by NTA using ZetaView PMX 110 and the corresponding software ZetaView v8.04.02. The system was calibrated and aligned with 102 nm polystyrene standard beads before the experiment. BEV samples were left at room temperature for 30 min to acclimatize before measurement. Samples were diluted to appropriate concentration (1:20,000 to 1:320,000) in ultrapure water to reach particle numbers ideal for NTA. All samples were analyzed under the same condition (temperature 25 °C, pH 5.8, sensitivity 92, shutter speed 70, and frame rate 30 fps). Triplicate measurements were taken for size and concentration, and each replicate included eleven positions with two cycles of reading at each position. The size distribution of BEVs was reported from 10 to 500 nm and the concentration was normalized to the volume of plasma and reported as particles per milliliter of blood plasma. For ζ potential measurement, data were acquired at least in quintuplicate and each replicate corresponded to two cycles of reading.

### 2.6. Western Blot Analysis of EV Cargo and Cellular Gene Expression

A total of 20 µg of BEVs or U937 protein extracts was subjected to 4–20% SDS-PAGE. The proteins were then transferred to a PVDF membrane, the membrane blocked with 5% BSA in 1× TBST buffer and incubated with relevant primary antibodies at 4 °C overnight. The blot was rinsed with 1× TBST 3–5 times for 5 min and incubated with relevant secondary antibodies for 1 h at room temperature. The blot was rinsed with 1× TBST 3–5 times for 5 min and images were captured using the Odyssey infrared imaging system (LI-COR). The integrated density of the band was measured using ImageJ 1.52a software.

### 2.7. Viability Assay

U937 cells were seeded (10,000 cells/well) atop a collagen-coated 96-well plate. Cells were treated with BEVs (concentrations, µg/mL, as shown on the figure) or an equivalent volume of 1× DPBS for 18 h at 37 °C. Cells were collected after treatment and tested for viability via the trypan blue exclusion assay. Cells were counted and viability determined using the Luna-II automated cell counter. 

### 2.8. Internalization of BEVs by Monocytes 

BEVs for each respective treatment were re-suspended in 100 µL of 1× DPBS. SYTO RNASelect stain stock solution was added to each 100 µL aliquot and mixed to obtain a final dye concentration of 10 μM. Samples were incubated at 37 °C for 20 min protected from light. Excess unincorporated dye was removed from the labeled BEVs with exosome spin columns (MW 3000) via centrifugation at 750× *g* for 2 min at room temperature. In total, 1000 µg/mL of labeled BEVs were added to U937 cells plated (10,000 cells/well) on a 96-well glass-bottom dish coated with type I collagen and incubated at 37 °C for 18 h, followed by a 5 min DAPI stain. Images were acquired using a Lionheart FX automated microscope. Representative 10× and 60× magnification images were acquired manually for five fields of view per well. Images were processed using Gen5 ImagePrime. The 10× magnification images were analyzed to quantify single-cell fluorescence intensity using Biotek’s Gen 5 software and raw data were plotted using GraphPad Prism 8 (La Jolla, CA, USA, www.graphpad.com).

### 2.9. Cell Spreading and Adhesion Assays

In total, 100,000 cells/mL of U937 monocytes were pre-treated with 1× DPBS, or BEVs (1000 μg/mL) from different clinical groups for 2 h at 37 °C. A total of 10,000 cells were then seeded in 96-well flat-bottom tissue culture plates pre-coated with type I collagen for 2 h at 37 °C. For the cell spreading assay, the plate was monitored through Lionheart FX automated microscope at 0 and 2 h time points. Representative 10× magnification brightfield images were captured for three fields of view per well. Quantification of cellular number, size and area was performed by Gen5 ImagePrime. For the cell adhesion assay, PBS- (1× DPBS) and BEV-treated cells were incubated atop collagen-coated wells for 18 h. Unbound cells were washed with 1× DPBS three times. Bound cells were stained with NucBlue cell stain for 20 min at room temperature. The whole well was captured through Lionheart FX automated microscope at 4× magnification. Quantification of cellular numbers was performed by Gen5 ImagePrime.

### 2.10. Analysis of Cytoskeletal Dynamics 

U937 cells (10,000 cells/well) were seeded in a 96-well glass-bottom dish coated with type I collagen and treated with 1000 µg/mL of respective BEV, or an equivalent volume of 1× DPBS and incubated at 37 °C for 18 h. Following incubation, cells were washed with 1× DPBS and fixed with 4% paraformaldehyde (PFA) in 1× DPBS for 15 min. Cells were then permeabilized by incubation in 0.1% Triton X-100 for 10 min. AlexaFluor 594 Phalloidin was applied in a 1:40 dilution for 1 h, followed by a 5 min DAPI stain. Images were acquired using a Lionheart FX automated microscope. Representative 10× and 60× magnification images were acquired manually for five fields of view per well. Image processing was performed using Gen5 ImagePrime. Quantification of F-actin levels, cell size, area, and circularity was performed by Gen5 ImagePrime via single-cell masking of phalloidin stain. A circularity metric was created by inputting the equation C = 4πA/P2, where C is the circularity, A is the area of the cell, and P is the perimeter of the cell. The site used in the analysis can be found following this link (https://imagej.nih.gov/ij/plugins/circularity.html). The 10× magnification images were utilized to analyze single cells using Biotek’s Gen 5 software and raw data were plotted using GraphPad Prism 8.

### 2.11. Statistical Analysis 

GraphPad Prism v 8.4.2 was used to plot all graphs and perform all statistical analysis. For two-group comparison, unpaired t test with Welch’s correction was used to determine the differences between the groups. Ordinary one-way ANOVA (Brown–Forsythe and Bartlett tests, with Sidak’s multiple comparisons test) was used to determine the differences between multiple groups. Binary Student’s *t* tests (Welch’s correction) were used to determine significant differences between groups for each of the time points in each group. 

## 3. Results

### 3.1. VEH/SIV and THC/SIV Rhesus Macaques Have Similar Blood Plasma Spectra

**Figure 1:**
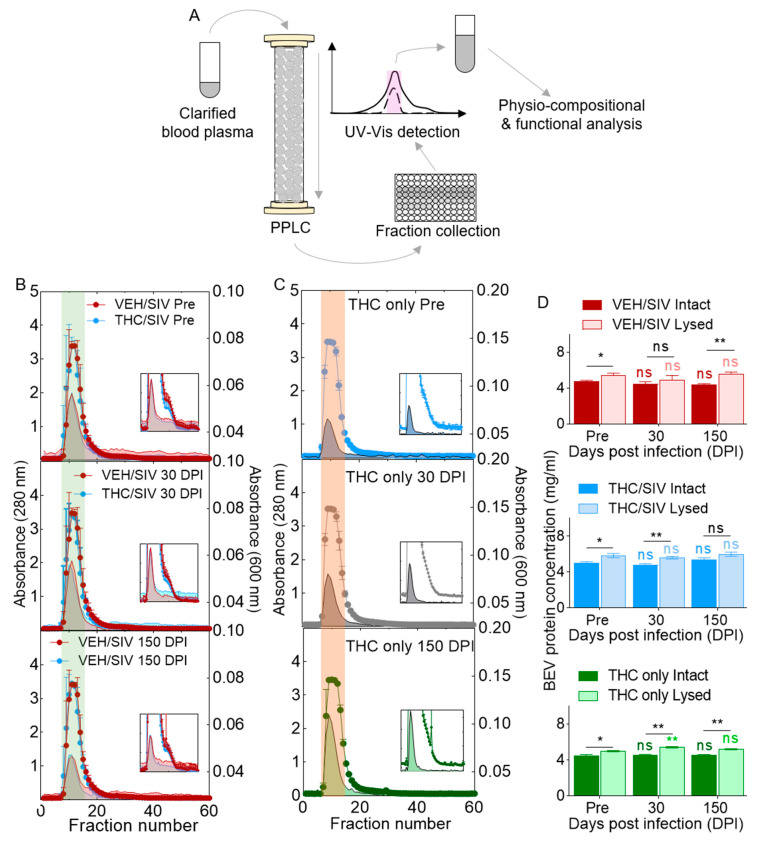
Isolation of BEVs from blood plasma of rhesus macaques (RM): (**A**) schematic of BEV isolation process. UV–vis absorbance profiles for the (**B**) VEH/SIV and THC/SIV groups, and the (**C**) THC-only group. (**D**) BEV protein levels for all clinical groups. Ordinary one-way ANOVA (Brown–Forsythe and Bartlett tests, with Sidak’s multiple comparisons test) was used to determine the statistical significance within the group. Binary Student’s *t* tests (Welch’s correction) were used to determine significant differences between groups for each of the time points in each group. ** *p* < 0.01, * *p* < 0.05, and ns = non-significant.

Clarified blood plasma is a mildly alkaline aqueous fluid containing water, clotting factors, electrolytes, hormones, antibodies, viral proteins, and genetic material. If not all, at least some of these factors are associated with EVs. Since we are analyzing longitudinal samples, it is important to monitor the isolation profiles for SIV- or THC-induced differences. To this end, we used a novel purification protocol—Particle Purification Liquid Chromatography (PPLC)—that we previously described [42] to (i) gain insight into total plasma spectra from the two clinical groups, and (ii) identify and collect pure EVs devoid of other factors. This purification process eliminates most albumin and non-membranous particles including exomeres and lipoproteins [63] that often times co-purify with EVs [64]. The schematic of BEV isolation and purification through PPLC is shown in Figure 1A. The elution profiles from VEH/SIV (*n* = 7) and THC/SIV (*n* = 7) groups are similar (Figure 1B, Figures S1 and S2). The 280 nm profiles (open histograms), which depict region of free protein [42], showed similar trends for both groups, which included a major peak (green highlight) from fraction 8 to 15 and a shoulder peak (insets) from fraction 16 to 25. The 600 nm profiles (close histograms), which mark regions enriched with EV, are also similar, but this profile excludes the second peak. BEVs were collected based on the merged peak region of 280 and 600 nm (green highlight). To ensure that THC treatment did not affect BEV profile, we also provided isolation profiles from RMs (*n* = 3) chronically (pre-SIV, 30, and 150 DPI) treated with THC that were not SIV infected (THC only). Similar profiles were obtained from the THC only group (Figure 1C, Figure S3). Analysis of intact BEV protein content showed no significant differences (range = 3.61 to 6.08 mg/mL) in intragroup protein concentration for the VEH/SIV and THC/SIV groups, with a subtle intragroup difference for the THC only group. Unlike the intact protein concentrations, intersample differences (range = 3.15 to 6.80 mg/mL) were observed in the three treatment groups, including at pre-SIV and 150 DPI for VEH/SIV, pre-SIV and 30 DPI for THC/SIV, and pre-SIV, 30, and 150 DPI for the THC only group (Figure 1D). 

### 3.2. Long-Term Low-Dose THC Treatment Decreased BEV Concentration in SIV-Infected RMs 

**Figure 2:**
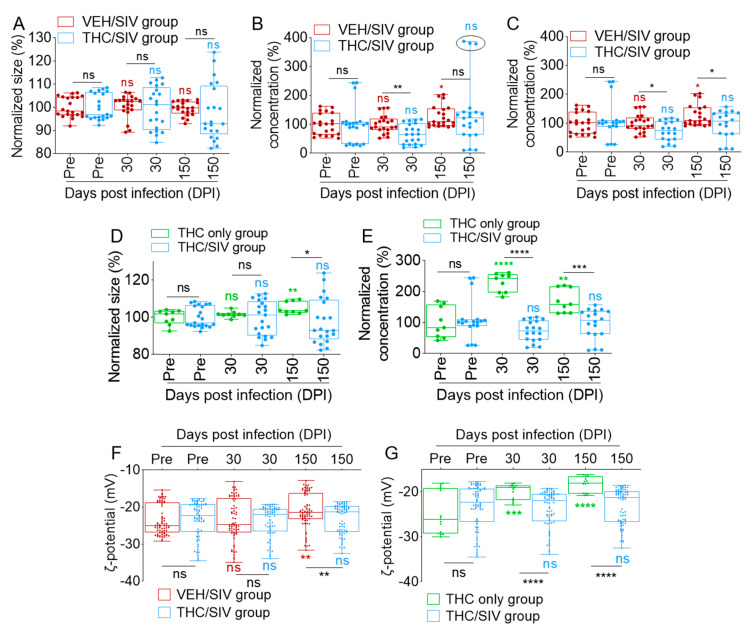
The effects of SIV infection and THC treatment of SIV-infected RMs on the physical properties of BEVs: (**A**) adjusted BEV size comparison between VEH/SIV and THC/SIV groups. (**B**) Adjusted BEV concentration comparison between the VEH/SIV and THC/SIV groups. (**C**) Adjusted BEV concentration comparison between the VEH/SIV and THC/SIV groups without the outlier THC/SIV RMs at 150 DPI (circle in B). (**D**) Adjusted BEV size comparison between the THC/SIV and THC only groups. (**E**) Adjusted BEV concentration comparison between the THC/SIV and THC only groups. (**F**) BEV ζ potential comparison between the VEH/SIV and THC/SIV groups. (**G**) BEV ζ potential comparison between the THC/SIV and THC only groups. For adjustments, the 30 and 150 DPI data were normalized according to respective pre-SIV data. Ordinary one-way ANOVA (Brown–Forsythe and Bartlett tests, with Sidak’s multiple comparisons test) was used to determine the statistical significance within the group. Binary Student’s *t* tests (Welch’s correction) were used to determine significant differences between groups for each of the time points in each group. **** *p* < 0.001, *** *p* < 0.005, ** *p* < 0.01, * *p* < 0.05, and ns = non-significant.

We have previously used different isolation protocols to purify EVs from both human semen and blood [34,35,36,37,38,39,40,41,42,44] and, recently, we optimized the isolation protocol that utilized a size-guided chromatographic technique to purify EVs from all body fluids [42]. We used this protocol to purify EVs from blood to gain novel insights into the effect of SIV infection and THC treatment on BEV size and concentration. Accordingly, we isolated BEVs from RMs pre-infection (pre-SIV, *n* = 14; VEH *n* = 7 and THC *n* = 7) and after (30 DPI, *n* = 7; and 150 DPI, *n* = 7) exposure to SIV and treatment with THC. As indicated in Figure S3A, the sizes (raw values) of BEVs from both the VEH/SIV and THC/SIV groups ranged from 103.9 to 125.8 nm. THC/SIV BEVs appeared smaller and showed significant size difference at *t* = pre-SIV (compared to VEH/SIV), with no difference at 30 and 150 DPI (Figure S4A). Given the group size difference at *t* = pre-SIV, we used the *t* = pre-SIV samples to adjust for background differences (details in methods section). The background-adjusted data show that although THC/SIV BEVs were a bit smaller, the difference did not reach statistical significance (Figure 2A).

Applying the same analytical protocol to BEV concentration, we found that the number of BEVs per mL of plasma was similar for both groups at pre-SIV with an increase in the VEH/SIV group at 150 DPI for both the raw (Figure S4B) and the background-adjusted (Figure 2B) concentrations. Interestingly, THC treatment significantly decreased BEV concentration at 30 DPI, with a non-significant decrease at 150 DPI (Figure S4B and Figure 2B). The absence of a significant decrease in the THC group may be attributed to one outlier in the group (Figure S4B and Figure 2B, circles on last bar). Removal of the outlier leaves six animals in the group and confirmed that chronic THC treatment resulted in a significant decrease in BEV concentration (Figure S4C and Figure 2C). 

To determine whether the changes in BEV size and concentration were related to THC treatment, we analyzed BEV size and concentration from the THC-only group. Unlike THC/SIV BEVs, THC-only BEVs showed a significant increase in raw and adjusted size at 150 DPI (Figure S4D and Figure 2D). With respect to concentration, THC/SIV BEVs did not change over time but THC only BEVs increased at 30 DPI and then decreased at 150 DPI (Figure S4E). Comparative analysis showed that THC significantly reduced the concentration of BEVs in SIV-infected RMs (Figure 2E). We also evaluated the electrostatic properties (measured as zeta (ζ) potential) of BEV membrane to assess SIV- and THC-induced changes. In general, RM BEVs bear negative (−23.56 ± 3.94 mV) surface charge and SIV infection significantly decreased the membrane charge from −23.39 ± 5.61mV at 30 DPI to −21.04 ± 4.67 mV at 150 DPI. At these time points, the surface charge of THC/SIV BEVs was −23.79 ± 4.11 mV at 30 DPI to −23.21 ± 3.95 mV at 150 DPI, suggesting that THC prevented a SIV-mediated decrease in BEV ζ potential (Figure 2F). Interestingly, chronic administration of THC alone significantly increased BEV ζ potential from −24.81 ± 4.45 mV at pre-SIV to −19.94 ± 1.64 mV at 30 DPI, and −18.35 ± 1.67 mV at 150 DPI (Figure 2G). These data suggest that the effect of THC on BEV ζ potential is distinct from that of SIV, and that both SIV infection and THC treatment may change the net charge on BEV membranes. 

Together, these data show that although individual variabilities exist as expected, adjustment for relative basal BEV size, concentration, and ζ potential prior to infection for individual subjects produced consistent results for the period of infection. Based on these findings, we focused on the VEH/SIV and THC/SIV groups in subsequent analysis. Raw data of individual RMs are presented in Figures S5–S7.

### 3.3. Infection Regulates the Levels of BEV-Associated Tetraspanins as Well as Other Protein Complexes

**Figure 3:**
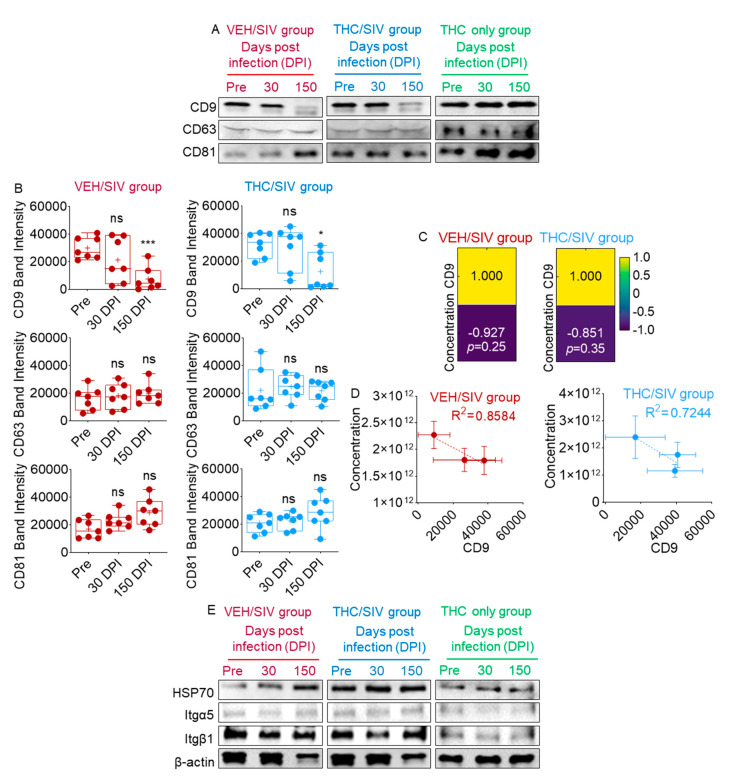
SIV infection and THC treatment of RMs induced changes in BEV-associated tetraspanins and other proteins: Western blot analysis of BEV markers—CD9, CD63, CD81—for (**A**) pooled BEV samples. (**B**) Densitometry of BEV markers for pre-SIV, 30 and 150 DPI for individual BEV samples of the VEH/SIV and THC/SIV groups (Figure S8). Ordinary one-way ANOVA (Brown–Forsythe and Bartlett tests, with Sidak’s multiple comparisons test) was used to determine the statistical significance within the group. *** *p* < 0.005, * *p* < 0.05, and ns = non-significant. (**C**) Pearson correlation analysis and (**D**) linear correlation analysis between BEV-associated CD9 and BEV concentration for the two groups. Scale bar represents the power of correlation between factors; a higher value represents higher correlation. (**E**) Western blot of HSP70, integrin protein and β-actin levels in pooled BEV samples.

Multiple families of proteins on producer cells, such as the membrane-spanning tetraspanins (CD9, CD63, and CD81), are used as markers of EVs. In addition to their role as EV markers, tetraspanins function as molecular scaffolds and distribute proteins into highly organized microdomains consisting of adhesion, signaling, and adaptor proteins. To evaluate the levels of EV markers and assess whether SIV infection or THC treatment affected EV markers, we examined the presence of EV markers on pooled RM BEVs using Western blotting. All three tetraspanins—CD9, CD63, and CD81—were present in BEVs irrespective of infection or treatment status (Figure 3A). However, there was a time-dependent decrease in the level of BEV-associated CD9 that was most prominent at 30 and 150 DPI in VEH/SIV BEVs compared to THC/SIV BEVs (Figure 3A). In contrast to CD9, CD63 was steady in all groups and at all time points. However, CD81 levels increased over time in the VEH/SIV group but not in the THC/SIV group (Figure 3A). Given the significant changes in BEV-associated CD9 and CD81 in the aggregate Western blot data, we next sought to understand these changes at the individual RM level. Figure S8 shows Western blot data on CD9, CD63, and CD81 from each of the 14 RMs. As indicated, all 14 RMs had little to no CD9 by 150 DPI, while 5 of 7 VEH/SIV and 2 of 7 THC/SIV RMs had reduced CD9 at 30 DPI. Additionally, 5 of 7 VEH/SIV and 3 of 7 THC/SIV RMs had increased CD81 at 150 DPI. Mean densitometry values for the tetraspanins in Figure S8 are shown in Figure 3B.

Because it has been suggested that in some cell line settings, CD9 overexpression enhances EV release [65], we examined inter-relationships between tetraspanin levels and BEV numbers as they relate to treatment groups. According to Pearson correlation analysis and linear correlation analysis, the decrease in CD9 abundance in BEVs was not associated with BEV concentration (Figure 3C,D). 

Further analysis of EV markers associated with VEH/SIV and THC/SIV BEVs showed that the level of the heat shock protein HSP70 mirrored that of CD81 (Figure 3A), where a time-dependent increase was observed in all groups, although more prominent in BEVs in the VEH/SIV group (Figure 3E). However, this trend was not observed in the THC-only group (Figure 3E).

Since tetraspanins regulate the integrin (Itg) family of proteins and tetraspanin•Itg complexes are known to regulate cell polarity [66,67], we examined the levels of Itg α5 and β1 in VEH/SIV and THC/SIV BEVs. The levels of Itg α5 and β1 were similar between groups, although there was a subtle decrease in Itg β1 levels in VEH/SIV BEVs (Figure 3E). In general, Itg α5 was lower in intensity compared to Itg β1. The level of β-actin was also examined. The intensity was similar between groups with a treatment-independent decrease in 150 DPI BEVs. Together, these findings imply that infection with SIV or chronic exposure to THC has distinct effects on BEV protein cargo. Remarkably, SIV infection decreased BEV-associated CD9 and increased CD81, while chronic THC exposure may reduce the numbers of CD81-associated vesicles in the blood of SIV-infected RMs, which may have functional effects on target cells. Indeed, tetraspanins, especially CD9 and CD81, are known to facilitate diverse fusion events, such as those that occur between gametes [68,69], myoblasts [70], or virus-infected cells [71,72], but inhibit fusion of mononuclear phagocytic cells [73]. 

### 3.4. Human U937 Monocyte-Like Cells Tolerate VEH/SIV and THC/SIV BEVs 

**Figure 4:**
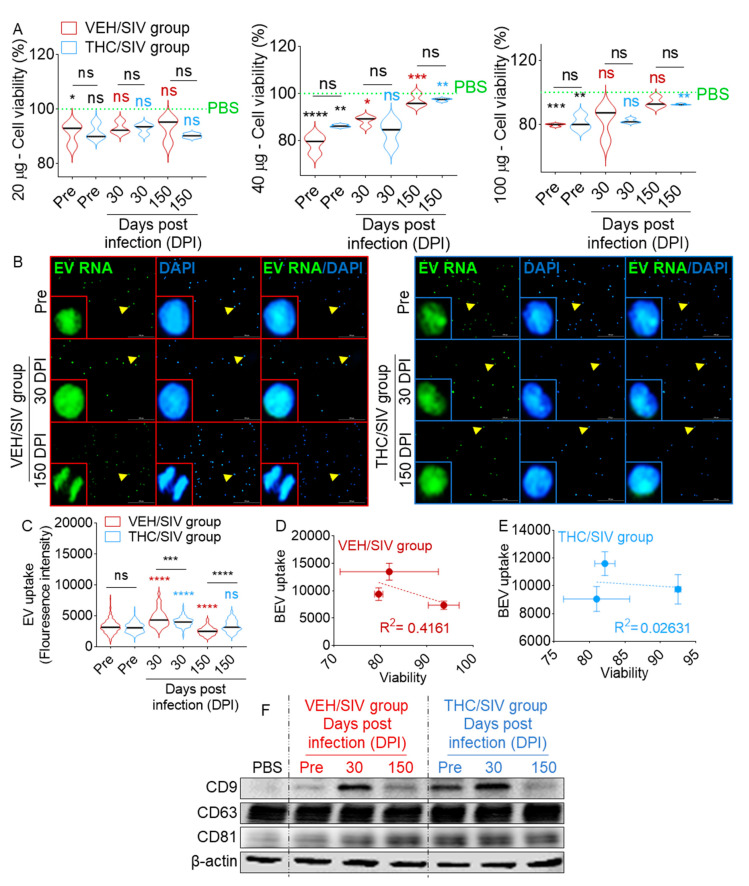
VEH/SIV- and THC/SIV-internalized BEVs transfer their cargo to target cells: (**A**) trypan blue exclusion assay for U937 following 18 h incubation with different concentrations (20, 40 and 100 µg) of BEVs. Ordinary one-way ANOVA (Brown–Forsythe and Bartlett tests, with Sidak’s multiple comparisons test) was used to determine the statistical significance within the group. Binary Student’s *t* tests (Welch’s correction) were used to determine significant differences between groups for each of the time points in each group. (**B**) Images of U937 cells incubated with SYTO RNASelect-stained BEVs (100 µg) for 18 h. DAPI is blue and SYTO RNASelect, which stained BEV RNA, is in green. Fluorescence images were manually obtained with Lionheart FX automated microscope at 10× magnification. Yellow arrows correspond to the enlarged area (inset). Scale bar: 50 μm. (**C**) Quantification of BEV internalization efficacy by U937 cells. Five fields of view were analyzed per time point. Ordinary one-way ANOVA (Brown–Forsythe and Bartlett tests, with Sidak’s multiple comparisons test) was used to determine the statistical significance within the group. Binary Student’s *t* tests (Welch’s correction) were used to determine significant differences between groups for each of the time points in each group. * *p* < 0.05, ** *p* < 0.01, *** *p* < 0.005, **** *p* < 0.001, and ns = non-significant. (**D**,**E**) Correlation analysis between cell viability and BEV internalization. (**F**) Western blot analysis of tetraspanins in U937 cells treated with VEH/SIV and THC/SIV BEVs; β-actin was used as a loading control.

Prior to conducting functional studies, we sought to evaluate the tolerance of BEVs by U937 monocytes by assessing cellular viability upon treatment with different concentrations (20, 40, and 100 µg) of BEVs. Cells seeded atop collagen-coated 96-well plates were treated with different concentrations of VEH/SIV or THC/SIV BEVs, while PBS-treated cells served as negative controls. After 18 h, cells were analyzed for viability using the trypan blue exclusion assay. In general, BEVs from SIV-uninfected THC-untreated (control) RMs had a concentration-dependent effect on monocyte viability (Figure 4A). Compared to the viability of PBS-treated cells that was set to 100%, BEVs from uninfected RMs significantly reduced monocyte viability (one-way ANOVA, Sidak’s multiple comparisons test) but there were no significant intergroup (VEH/SIV vs. THC/SIV, unpaired t test with Welch’s correction) differences (Figure 4A and Table 3).

Next, we evaluated the effect of BEVs from 30 and 150 DPI RMs on cell viability. In comparison to pre-SIV BEVs set at 100% for each concentration in each group. A total of 20 µg BEVs from both groups showed no significant change in cell viability (one-way ANOVA, Sidak’s multiple comparisons test). However, at 40 µg, both VEH/SIV and THC/SIV BEVs increased cell viability relative to control BEVs, although the change in 30 DPI THC/SIV was not significant (Figure 4A, middle panel). At 100 µg, there was a subtle non-significant increase in the viability of cells treated with VEH/SIV and THC/SIV BEVs compared to control BEVs set at 100% (one-way ANOVA, Sidak’s multiple comparisons test), except for THC/SIV at 150 DPI (*p* = 0.0051, Figure 4A, bottom panel). Analysis of intergroup differences (unpaired t test with Welch’s correction) showed no significant differences in the viability of cells treated with VEH/SIV or THC/SIV. These data suggest that BEVs reduce monocyte viability independent of SIV infection or THC treatment. Since both 40 and 100 µg BEV concentrations have similar effects on cell viability, we used 100 µg of BEVs in subsequent studies to ensure robust BEV-to-cell interaction and avoid potential bystander effects. 

### 3.5. VEH/SIV and THC/SIV BEVs are Internalized by Human U937 Monocyte-Like Cells and the BEVs Modulate Cellular Gene Expression

Equal concentrations of VEH/SIV and THC/SIV BEVs were pre-stained with SYTO RNASelect stain, a green fluorescent stain selective for RNA (absorption/emission maxima of 490/530 nm). Following removal of unincorporated dye via exosome spin columns, labeled BEVs were added to U937 cells atop collagen-coated 96-well glass-bottom dish. Approximately 18 h later, single-cell BEV internalization was analyzed microscopically, and images were processed with Gen5 ImagePrime software. Clusters of labeled control, VEH/SIV, and THC/SIV BEVs containing RNA are abundant in the cytosol and nucleus of the cells, indicating that the cells readily internalized BEVs [74] and transferred their RNA cargo to cells (Figure 4B). The relative internalization (single-cell fluorescence intensity of SYTO RNASelect stain) of BEVs for both the VEH/SIV and THC/SIV groups were higher at 30 DPI compared to 150 DPI (Figure 4C). Intergroup differences in BEV internalization was observed, where internalization of 30 DPI THC/SIV BEV was lower (*p* = 0.0009) compared to VEH/SIV BEV. In contrast, internalization of 150 DPI THC/SIV BEV was higher (*p* = <0.0001) compared to a similar time point for VEH/SIV BEV. Noteworthy, internalization of BEVs was determined via single-cell analysis of SYTO RNAselect fluorescence intensity, eliminating the possibility that differences in BEV internalization were the result of differences in viability, as only viable cells were analyzed. Absence of correlation between BEV internalization and cell viability (Figure 4D,E) further validates this point. Intergroup variation in BEV internalization may be due to varying RNA content in the BEVs, since the readout for internalization was the analysis of BEV RNA cargo via SYTO RNASelect stain. This observation is not surprising, since it has been shown that BEVs derived from serum contain an extremely diverse RNA cargo, with some containing substantial numbers of RNA molecules, while others contain little to no RNA [74]. 

In addition to the transfer of BEV RNA to cells, we observed changes in the protein levels of select genes in cells treated with the different BEVs. U937 cells contain very low levels of CD9 [75], as shown in Figure 4F (PBS lane). Interestingly, an increase in CD9 and CD81 intensities was observed in cells treated with control, VEH/SIV, and THC/SIV BEVs, although at varying degrees (Figure 4F).

### 3.6. VEH/SIV BEVs Potentiate BEV-Mediated Human U937 Monocyte-Like Cell Spreading and Adhesion to Type I Collagen While THC/SIV BEVs dampen the effect

**Figure 5:**
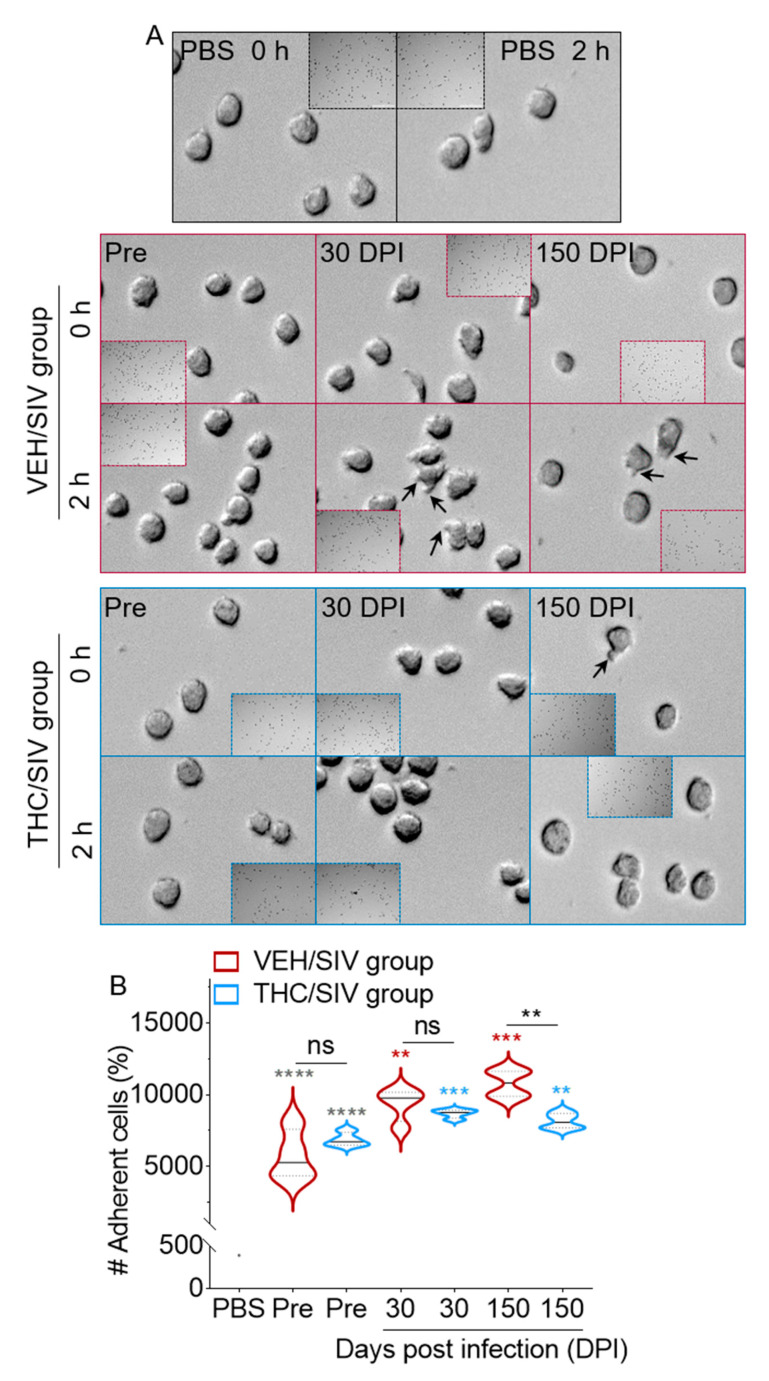
U937 spreading and adhesion to type I collagen are inhibited by THC/SIV BEVs: (**A**) representative images of U937 cell spreading on type I collagen following treatment with BEVs at 1000 µg/mL. The 10× magnification kinetic images were acquired at *t* = 0 h and *t* = 2 h using the Lionheart FX automated microscope. Black arrows indicate membrane protrusions. Scale bar: 50 μm. (**B**) Quantification of adherent monocytes incubated with 1000 µg/mL BEVs for 18 h. Full view of each well was analyzed. Ordinary one-way ANOVA (Brown–Forsythe and Bartlett tests, with Sidak’s multiple comparisons test) was used to determine the statistical significance within the group. Binary Student’s *t* tests (Welch’s correction) were used to determine significant differences between groups for each of the time points in each group. ** *p* < 0.01, *** *p* < 0.005, **** *p* < 0.001, and ns = non-significant.

For BEVs to be internalized into cells, deliver their cargo, and/or stimulate recipient cells, they must interact with the cell membrane. Thus, we examined the effect of VEH/SIV and THC/SIV BEVs on the morphology and cytoskeletal dynamics of monocytes. Monocytes, either pre-treated or not with control, VEH/SIV, and THC/SIV BEVs were assessed for their ability to spread on collagen-coated coverslips over time. After 2 h of incubation on coated coverslips, PBS did not significantly change monocyte spreading (Figure 5A, top panel). However, VEH/SIV BEV-treated cells began spreading by extending cytoplasmic processes that were clearly detectable in 30 and 150 DPI BEV-treated cells (Figure 5A, middle panel). In contrast, THC/SIV BEV-treated cells produced minimal cytoplasmic extensions at all time points measured (Figure 5A, bottom panel). These data suggest that spreading cells may use their extensions to contact the ECM and form adhesions. PBS-treated cells exhibit minimal adhesion in the absence of BEVs and were used as background. Treatment of cells with pre-SIV BEVs from both groups resulted in a time-dependent increase in adhesion compared to PBS, with no difference between the groups (Figure 5B). Optimal adhesion to collagen was observed when cells were treated with VEH/SIV and THC/SIV BEVs (Figure 5B). Intergroup comparison showed that the THC/SIV BEVs at 150 DPI significantly suppressed cellular adhesion to collagen compared to those from VEH/SIV (Figure 5B), suggesting that THC treatment may alter the composition of EVs in such a way that they reduce adhesion of U937 cells and, by extension, monocyte adhesion to the vascular endothelial lining and subsequent migration and extravasation. A similar observation was made by other investigators, where THC decreased Tat-induced U937 adhesion [31]. 

### 3.7. VEH/SIV and THC/SIV BEVs Induce Distinct Cytoskeletal Changes in Human U937 Monocyte-Like Cells

**Figure 6:**
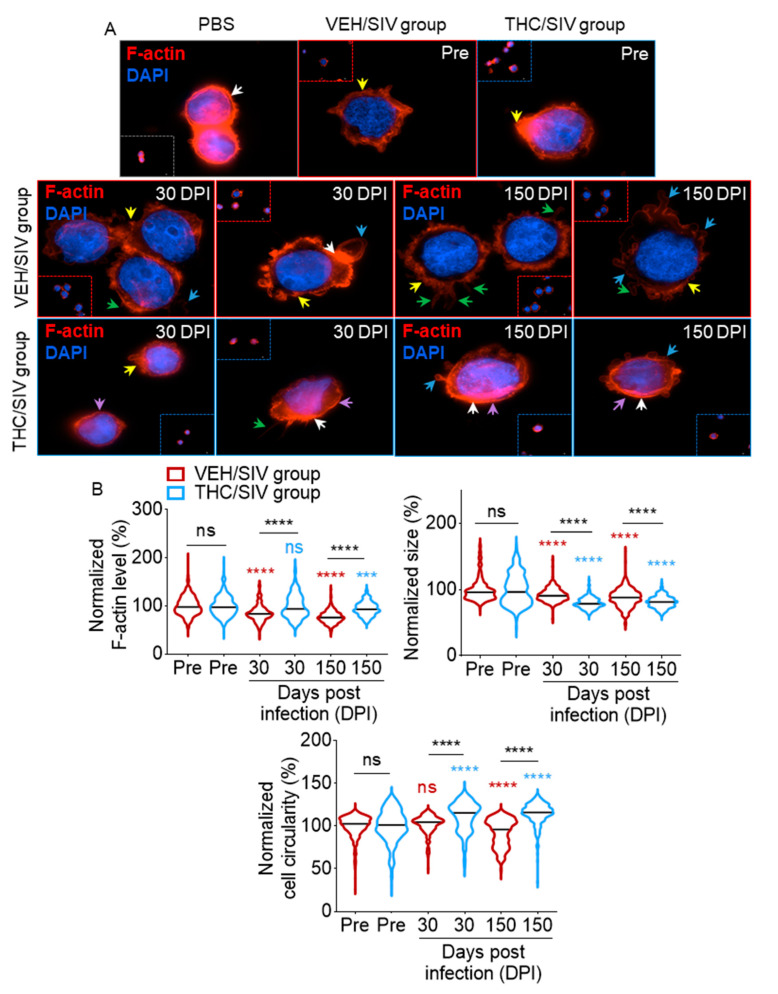
THC/SIV BEVs dampen SIV-induced changes in cytoskeletal organization: (**A**) F-actin (red) and DAPI (blue) staining of U937 cells incubated with 1000 µg/mL BEVs for 18 h. Fluorescence images were manually obtained at 60× magnification using the Lionheart FX automated microscope. Yellow arrows depict membrane ruffling, white arrows depict areas of increased F-actin localization, green arrows depict filopodia-like protrusions, blue arrows depict lamellipodia-like protrusions, and purple arrows depict smooth prominent cortical shell. (**B**) Quantification of single-cell F-actin fluorescence intensity (top left panel), cell size (top right panel), and cell circularity (bottom panel). Five 10× magnification fields of view were analyzed per time point. Ordinary one-way ANOVA (Brown–Forsythe and Bartlett tests, with Sidak’s multiple comparisons test) was used to determine the statistical significance within the group. Binary Student’s *t* tests (Welch’s correction) were used to determine significant differences between groups for each of the time points in each group. *** *p* < 0.005, **** *p* < 0.001, and ns = non-significant.

Since BEVs affected cell adhesion and spreading, we assessed the effect of BEVs on the actin cytoskeleton. At 18 h post-treatment, PBS-treated monocytes showed maintenance of cortical actin filaments, with discrete deformations in the actin cytoskeleton caused by adherence to collagen (Figure 6A, white arrows in top left panel). Incubation of U937 monocytes with BEVs isolated from both groups at the pre-SIV time point induced actin cytoskeletal rearrangements, consisting of a loss of rounded shape and areas of increased F-actin intensity (yellow arrows), indicative of monocyte polarization [76,77]. Treatment with VEH/SIV BEVs from both 30 and 150 DPI (Figure 6A, middle panel) induced the formation of pronounced lamellipodia- (blue arrows) and filopodia-like extensions (green arrows), with extensive presence of membrane ruffling (yellow arrows). In contrast, THC dampened the effect of SIV (VEH/SIV), since cells treated with THC/SIV BEVs exhibited reduced membrane ruffling, filopodial and lamellipodial protrusions (Figure 6A, bottom panel). THC/SIV BEV-treated cells also exhibit smooth prominent cortical shells (Figure 6A, bottom panel—purple arrows), which was rare in VEH/SIV-treated cells. Quantitative data analysis showed that treatment with VEH/SIV BEVs induced a significant decrease in F-actin polymerization at both 30 and 150 DPI (Figure 6B, top left panel, red violin plot) compared to the pre-SIV BEVs. The effect of THC is highlighted when comparing VEH/SIV BEVs to THC/SIV BEVs at 30 and 150 DPI, with both time points showing a THC-dependent attenuation of SIV-induced F-actin depolymerization (Figure 6B, top left panel, blue violin plot).

Additional morphometric analysis showed that at 18 h post-treatment, cells treated with pre-SIV BEVs had sizes that ranged from 16.56 to 19.3 μm (Figure 6B, top right panel). Relative cell size following adjustment with pre-SIV showed that at 30 and 150 DPI, THC/SIV BEVs significantly decreased monocyte size to an average size of 79.7 at 30 DPI and 81.2 at 150 DPI compared to 92.1 at 30 DPI to 87.5 at 150 DPI for cells treated with VEH/SIV BEVs (Figure 6B, top right panel). Furthermore, the cell membrane was traced and monocyte circularity was determined. Monocytes treated with 30 DPI VEH/SIV BEVs showed no significant change in circularity, while 150 DPI VEH/SIV BEVs displayed a significant decrease in cell circularity (Figure 6B, bottom panel). In contrast, THC/SIV BEVs induced a significant increase in monocyte circularity at both 30 and 150 DPI relative to VEH/SIV BEVs (Figure 6B, bottom panel). An increase in monocyte circularity suggests that THC/SIV BEVs induced a significant reduction in actin-rich membrane ruffles, lamellipodia- and filopodia-like protrusions. These results confirm that VEH/SIV and THC/SIV BEVs are functionally different, and that THC/SIV BEVs have the potential to modulate the organization of actin cytoskeleton induced by SIV during the processes of monocyte spreading.

### 3.8. VEH/SIV and THC/SIV BEVs Mediate Divergent Signaling in Human U937 Monocyte-Like Cells

**Figure 7:**
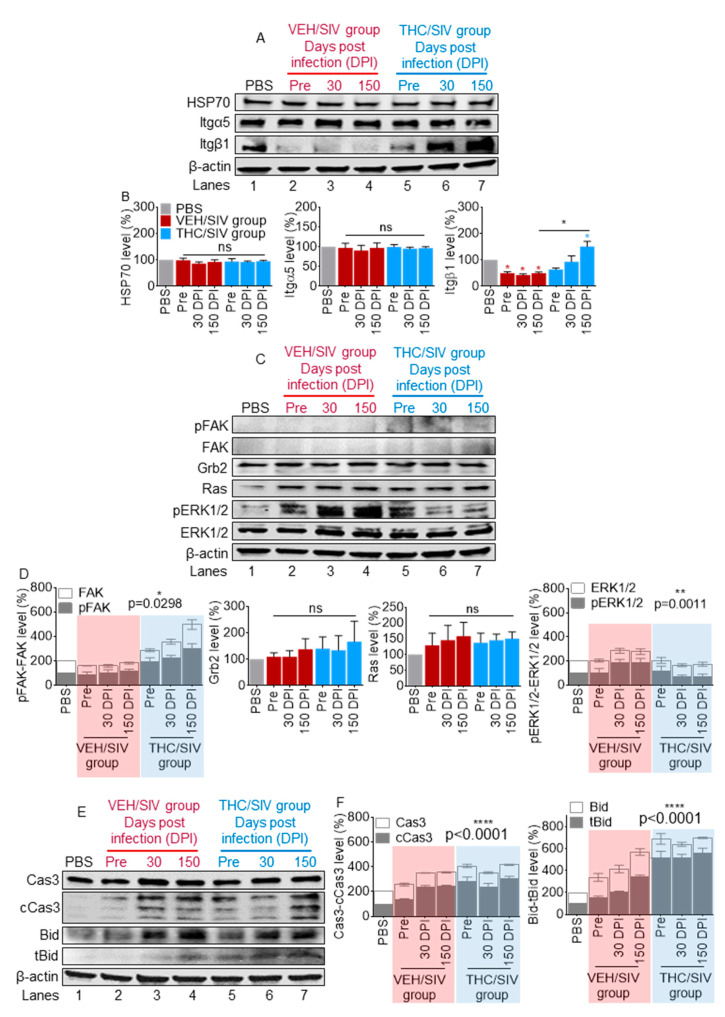
THC/SIV BEVs dampen SIV-induced changes in intracellular signaling: (A,B) representative Western blot images of HSP70, Itg α5 and Itg β1 (**A**) and densitometry quantification of three independent repeats (**B**). (C,D) Representative Western blot of kinases, their activators, and adaptor (**C**) and densitometry quantification of three independent repeats (**D**). (E,F) Representative levels of proteins linked to cell death (**E**) and densitometry quantification of three independent repeats (**F**). The relative band density was determined by normalizing to β-actin and PBS control. Ordinary one-way ANOVA (Brown–Forsythe and Bartlett tests, with Sidak’s multiple comparisons test) was used to determine the statistical significance within the group. Two-way ANOVA (groups x proteins) was used to determine the statistical significance for pFAK/FAK, pERK1/2/ERK1/2, cCas3/Cas3 and tBid/Bid. * *p* < 0.05, ** *p* < 0.01, **** *p* < 0.001, and ns = non-significant.

Given the different effects that VEH/SIV and THC/SIV BEVs exhibited in their adhesive functions, and the fact that integrins which regulate adhesion and interact with tetraspanins are present in BEVs, we next evaluated the ability of the BEVs to regulate intracellular signaling. While there was no change in the expression of Itg α5, we observed a strong upregulation of Itg β1 in THC/SIV-treated cells compared to those treated with VEH/SIV BEVs (Figure 7A,B). Binding of integrins to ECM proteins is known to induce downstream signaling. Thus, we assessed the phosphorylation of kinases FAK and ERK1/2. As expected, the level of FAK in U937 is low [78,79]. However, the levels of total FAK and phospho-FAK (Tyr397, pY397 FAK) were elevated in cells treated with THC/SIV BEVs (Figure 7C,D). Interestingly, pY397 FAK and ERK1/2 were found to be regulated in opposing ways by VEH/SIV and THC/SIV BEVs (Figure 7C,D). We observed a biphasic change in pY397 FAK and a significant reduction in pERK1/2 when monocytes were cultured with THC/SIV BEVs (lanes 6 and 7) compared to VEH/SIV BEVs (Lanes 3 and 4). However, treatment with both BEVs led to a significant induction of membrane-bound GTPase, Ras with no intergroup differences and no change in growth factor receptor-bound protein 2 (Grb2), a key adaptor protein that maintains Ras and ERK activity [80,81] (Figure 7C,D). These data strongly indicate that FAK and FAK signaling, along with ERK1/2 signaling, may be involved in the regulation of cell responses to THC/SIV BEVs.

Because cell adhesion to ECM substrates such as collagen generates transmembrane signals that regulate cell survival, we sought to further understand the effect of BEVs on molecular signals linked to cell death. Using Western blot analysis, we found that, in general, U937 cells treated with BEVs showed caspase 3 (Cas3) activation (Figure 7E,F). However, cells treated with VEH/SIV BEVs showed increased expression and cleavage of Cas3 and absence of the proapoptotic truncated Bid, tBid. In contrast, cells treated with THC/SIV BEVs showed no change in steady state levels of Cas3, or biphasic change in cleavage of Cas3, leading to a time-dependent increase in tBid. This observation suggests that THC/SIV treatment may induce apoptosis, since tBid translocation to the mitochondria promotes the oligomerization of Bax/Bak, facilitating the induction of cell death [82].

## 4. Discussion

In the present study, we provide novel data demonstrating that SIV infection alone and THC treatment of SIV-infected RMs result in the release of bioactive BEVs with potential to induce distinct cellular structural adaptations and signaling cues. The key observations from this study are that (i) BEVs released by SIV-infected RMs (VEH/SIV BEVs) may mediate pathogenic processes; and (ii) chronic exposure of SIV-infected RMs to THC (THC/SIV) results in the release of BEVs that dampen the ability of SIV infection to mediate cell spreading and adhesion on the ECM substrate collagen, alter cytoskeletal dynamics, and signal transduction—all of which may possibly instruct reductions in immune cell adhesion to vascular endothelium and extravasation as part of cannabinoid’s anti-inflammatory response to infection. The findings of the present study underscore the narrative that EV-mediated cellular communication and modulation of cellular function are at play in many physiological and pathological conditions. EVs carry out their functions through their bioactive cargo, which they deliver to target cells [35,38,83,84], as demonstrated by our findings that BEV RNA cargo from all clinical groups were successfully delivered to U937 cells. The physical and molecular composition of EVs is determined by the status of the producer cells, including producer cell origin, environmental conditions, and clinical status. Thus, accumulated evidence, including studies from our group [41,43], has shown that the functions of EVs vary and depend on the molecular composition of their cargo, as determined by the producer cells. These observations were further echoed in the present study because VEH/SIV BEVs were significantly different from THC/SIV BEVs in many aspects, including concentration, size, ζ potential, cargo composition, and function. 

Numerous studies, including studies from our group [8,9,10,11,12], showed that administration of THC is linked to a beneficial reduction in systemic inflammation and immune activation in ART-treated HIV+ individuals. In the SIV/rhesus macaque model, THC was shown to ameliorate SIV disease progression [24,25], reduce intestinal T cell activation/exhaustion and prevent lymph node fibrosis [10]. The benefits of THC are systemic, affect many organs, and are also present in BEVs, which may mediate THC action, as shown by the results of the present study.

Across all study subjects treated with THC, we found that THC treatment significantly inhibited BEV release and also reduced BEV size, albeit more variably. The effect of THC on BEV size and concentration was strictly dependent on the clinical status of the RMs because chronic exposure of SIV-uninfected RMs to THC resulted in the release of more BEVs that were also larger in size. This observation is in line with studies that showed that CBD is a potent inhibitor of EV release from different pathologic models, including prostate cancer, hepatocellular carcinoma, and breast adenocarcinoma cell lines [85]. This novel function of THC on BEV release, as observed in this study, may be of relevance for BEV-mediated modulation of HIV/SIV-induced pathologies, including chronic inflammation and cell activation. 

Of note is the strength of our longitudinal experimental design and collection of BEVs. The design allowed us to identify spaciotemporal differences in VEH/SIV BEVs and THC/SIV BEVs with regards to morphometric and cytoskeletal rearrangements as well as F-actin depolymerization. It is recognized that the cytoskeleton provides a scaffold for the plasma membrane cellular receptors to interact with the extracellular environment. However, pathogenic viruses such as HIV [86] manipulate the actin cytoskeleton in many ways [87]. Depending on the cell type, HIV-mediated changes in cellular morphology may result in increased viral spread and impairment of immune function [88]. Indeed, EV-associated HIV Nef is known to decrease Cdc42 activation, reduce actin polymerization, and increase lipid raft abundance [89,90,91]. In our studies, we found that Ras was induced by all BEVs irrespective of infection or treatment status. However, VEH/SIV BEVs decreased the level of F-actin, an effect that was not observed in THC/SIV BEV-treated cells, suggesting that THC treatment may overcome the reduction in F-actin abundance. In addition to F-actin levels, VEH/SIV BEVs decreased monocyte circularity but THC/SIV BEVs maintained monocyte circularity. The decrease in circularity suggests changes in cortical F-actin structures. Such protrusions may mediate cell adhesion and migration, since formation of filopodia-like protrusions has been implicated in myeloid cell migration and invasion through the ECM [92]. Our findings are remarkable because it has been shown that cortical actin depolymerization allows passage of the viral core through the dense actin cortex during inbound HIV infection [88]. Indeed, HIV Tat has been shown to modulate the expression of numerous genes involved in actin regulation, increasing cell motility, chemotaxis, transendothelial migration as well as membrane projections in monocytes [31,93]. Increased tissue infiltration of monocytes has also been observed in HIV+ individuals in vivo [94]. Moreover, CB2R activation has been shown to decrease monocyte chemotactic abilities and result in diminished lamellipodia formation [95]. These findings provide deeper insights into the extracellular mechanisms of THC action and may have broader implications for the clinical management of cardiovascular comorbidities such as atherosclerosis in HIV patients, where monocyte adhesion to the endothelium and migration are important initial steps in atherosclerotic plaque development [96].

It was suggested that EV-associated Nef potentiation of inflammatory responses may occur via the activation of ERK1/2 [91]. In our studies, we observed that THC/SIV BEVs downregulated pERK1/2, while upregulating pFAK and Itg β1. Indeed, integrins form complexes with tetraspanins, and tetraspanins may modulate the adhesive functions of integrins. Activation of integrins by ECM proteins leads to changes in protein tyrosine kinases, FAK and ERK1/2, which are downstream targets for integrin-initiated signaling [97,98,99]. The link between integrins and ERK is dependent on the integrity of the actin cytoskeleton. This link may be the activation of Rho and the Ras families of small GTPase proteins [100,101,102], and BEVs uniformly induced Ras expression in our study, even though there were apparent differences in ERK1/2 activation.

The role of integrins in biological interactions, such as cell–matrix or EV–matrix adhesion, is a three-step mechanochemical process, in which (i) ECM–integrin linkages need to withstand the forces at adhesion sites [103,104], (ii) the adhesion forces are translated into biochemical signals (mechanotransduction), and (iii) integrins mechanically link the actin cytoskeleton to transmit forces to the cells. In our system, although β1 integrin, FAK, pY397 FAK, and F-actin levels were high in cells treated with THC/SIV BEVs, there was a reduced integrated cellular response to adhesion. The association of THC to reduced adhesive response by monocytes has been demonstrated previously [31]. It has been shown that Tat increased U937 monocyte adhesion to ECM proteins through alteration in Itg β1 expression and distribution of polymerized actin. However, the effect of Tat was inhibited by THC and CP 55940 [31]. In our study, THC/SIV BEVs decreased U937 adhesion to collagen in contrast to the potentiation of adhesion by VEH/SIV BEVs. Interestingly, a THC/SIV-mediated decrease in cell adhesion correlated with decreased cell spreading on collagen, increased activities of the proapoptotic BH3-only member of the Bcl2 family of apoptotic proteins Bid and elevated expression of Cas3 cleavage products p19, p17 and p12. Whether or not loss of adhesion, Cas3 and Bid expression are regulated together or independently is yet to be determined, but it is noteworthy that the effect of THC was prominent with 150 DPI BEVs. Finally, the time-dependent increase in tBid expression in U937 cells is an important finding, as cannabinoids have been reported to exert immunosuppressive effects through induction of apoptosis in different immune cell populations [105]. These findings also suggest that THC and, by extension, other cannabinoids may exert their anti-inflammatory effects systemically, both in a paracrine and endocrine fashion, through the stimulated release of proapoptotic EVs very early in HIV/SIV infection. 

Our results suggest that the mechanochemical crosstalk between integrin–cytoskeletal adhesion as mediated by BEVs may have opposing roles in HIV/SIV infection. The ECM provides structural support for the invasion of HIV/SIV into various tissues, which involves pathologic adhesion and subsequent migration of virally-infected cells and inflammatory mediators across various blood–tissue barriers [106,107]. In this case, integrins are ECM-activated factors that provide anchorage to cells and are involved in the bidirectional interaction between cells, the actin cytoskeleton, and the ECM. Such interactions provide focal complexes, force transmission through the actin cytoskeleton and maturation of focal complexes to focal adhesions (FAs) [108,109,110]. In our studies, we found that, unlike VEH/SIV, THC/SIV BEVs upregulated F-actin, integrin β_1_, and pFAK, while decreasing cell adhesion. Although stable adhesion requires an Itg α5β1-mediated molecular bond or other Itg heterodimeric complexes, we found that the level of Itg α5 did not change between VEH/SIV BEVs and THC/SIV BEV-treated cells or even the EVs, which also contained Itgs. 

Binding of integrins to ECM proteins is known to induce downstream signaling. In our studies, we observed that elevated Itg β1 did not change the levels of HSP70, a family of molecular chaperones that protect cells from stress, regulate cell adhesion and invasion through the modulation of integrins β1, β2, and β3 and integrin-associated signaling molecules [111]. HSP70 is also known to prevent or arrest inflammation, and HSPs, in general, have been shown to promote the production of anti-inflammatory cytokines [112].

## 5. Conclusions

Our findings that THC/SIV BEVs induce F-actin, Itg β1, and FAK/pY397 FAK suggests activation of a mechanosensory-immunoregulatory pathway and function by THC-associated BEVs in HIV/SIV infection. This function is exemplified by the fact that the BEVs decreased cell adhesion in an environment with high levels of F-actin, Itg β1, and FAK/pY397 FAK. It is possible that reduced cell adhesion may limit the migration and infiltration of inflammatory cells into tissues as part of THC’s anti-inflammatory role. Future studies should investigate how mechanochemical events induced by BEVs influence actin, cytoskeleton, Itg β1, and FAK and their ability to regulate mechanotransduction. Finally, the mechanisms by which THC/SIV BEVs home to infected/inflamed sites should be investigated to provide insight into the rational design of targeted therapies to control inflammation.

## Figures and Tables

**Table 1:** Animal IDs, SIV inoculum, duration of infection and plasma viral loads in vehicle- or delta-9-tetrahydrocannabinol (Δ^9^-THC)-treated chronic SIV-infected rhesus macaques.

Animal ID	SIV Inoculum	Duration of Infection	Plasma Viral Loads 106/mL at 6 Months Post-SIV	Opportunistic Infections
Vehicle-treated SIV-infected rhesus macaques				
IH96	SIVmac251	180	0.1	ND
HV48	SIVmac251	150	4	ND
IN24	SIVmac251	180	9.4	ND
JC81	SIVmac251	180	0.38	ND
JH47	SIVmac251	180	2	ND
JR36	SIVmac251	180	0.5	ND
IV95	SIVmac251	180	0.02	ND
Delta-9-tetrahydrocannabinol-treated SIV-infected rhesus macaques				
IA83	SIVmac251	180	1.5	ND
IH69	SIVmac251	180	0.06	ND
HI09	SIVmac251	180	0.01	ND
JB82	SIVmac251	180	7.7	ND
IA04	SIVmac251	150	0.66	ND
JI45	SIVmac251	180	3	ND
JC85	SIVmac251	180	0.02	ND
Delta-9-tetrahydrocannabinol-treated SIV-uninfected rhesus macaques				
HN79	NA	NA	NA	NA
HN39	NA	NA	NA	NA
HI78	NA	NA	NA	NA

NA—not applicable; ND—none detected.

**Table 2:** Chemical reagents and instrument used in this study.

Chemicals	Company	City, State, Country	CatLog Number
Roswell Park Memorial Institute (RPMI) 1640	Corning	Corning, NY, USA	10-040-CV
Fetal bovine serum (FBS)	Atlanta Biologicals	Flowery Branch, GA, USA	S11150
Penicillin-streptomycin	Corning	Corning, NY, USA	30-002-CI
Amphotericin B	Corning	Corning, NY, USA	30-003-CF
Sodium pyruvate	Corning	Corning, NY, USA	25-005-CI
l-glutamate	Corning	Corning, NY, USA	25030081
4-(2-hydroxyethyl)-1-piperazineethanesulfonic acid (HEPES)	Research Products International	Mt Prospect, IL, USA	30TY40
Type I collagen, bovine	Corning	Corning, NY, USA	354231
10× DPBS	Corning	Corning, NY, USA	20-031-CV
Bradford reagent	Bio-Rad	Hercules, CA, USA	5000006
Trypan blue	Life Technologies	Carlsbad, CA, USA	15250061
SYTO RNASelect stain	Thermofisher	Grand Island, NY, USA	S32703
AlexaFluor 594 Phalloidin	Thermofisher	Grand Island, NY, USA	A12381
Triton X-100	Sigma	St. Louis, MO, USA	T8532
Paraformaldehyde (PFA)	Sigma	St. Louis, MO, USA	P6148
NucBlue™ Live ReadyProbes™ reagent	Thermo Fisher Scientific	Waltham, MA, USA	R37605
CD63 (H5C6)	Developmental Studies Hybridoma Bank (DSHB)	Iowa City, IA, USA	P08962
CD9 (602.29 cl. 11)	Developmental Studies Hybridoma Bank (DSHB)	Iowa City, IA, USA	P21926
CD81	Novus Biologicals	Centennial, CO, USA	SN206-01
HSP70	R&D systems	Minneapolis, MN, USA	AF1663
β-actin	Proteintech	Rosemont, IL, USA	60008-1-Ig
Integrin β1 (D2E5)	Cell Signaling	Beverly, MA, USA	9699
Integrin α5 (D7B7G)	Cell Signaling	Beverly, MA, USA	98204
Phospho-FAK (Tyr397) (D20B1)	Cell Signaling	Beverly, MA, USA	8556
FAK	Cell Signaling	Beverly, MA, USA	3285
Phosphor-ERK1/2 (pMAPK) (Thr202/Tyr204) (197G2)	Cell Signaling	Beverly, MA, USA	4377
P44/42 MAPK (ERK1/2) (137F5)	Cell Signaling	Beverly, MA, USA	4695
Cleaved caspase 3 (cCas3) (Asp175) (5A1E)	Cell Signaling	Beverly, MA, USA	9664
Caspase 3 (Cas3) (D3R6Y)	Cell Signaling	Beverly, MA, USA	14220
Bid/tBid (human specific)	Cell Signaling	Beverly, MA, USA	2002
Ras (27H5)	Cell Signaling	Beverly, MA, USA	3339
Grb2	Cell Signaling	Beverly, MA, USA	3972
IRDye^®^ 800CW donkey anti-mouse IgG (H + L)	LI-COR	Lincoln, NE, USA	926-32212
IRDye^®^ 800CW donkey anti-rabbit IgG (H + L)	LI-COR	Lincoln, NE, USA	926-32213
IRDye^®^ 680RD donkey anti-mouse IgG (H + L)	LI-COR	Lincoln, NE, USA	926-68072
Exosome spin columns	Thermofisher	Grand Island, NY, USA	4484449
96-well glass-bottom plate	Cellvis	Mountain View, CA, USA	P96-1.5P
PVDF membrane	Bio-Rad	Hercules, CA, USA	1620177
Sephadex G-50 fine beads	GE-Healthcare	Pittsburgh, PA, USA	17004201
Econo-column	Bio-Rad	Hercules, CA, USA	7374721
Synergy-H1 microplate reader	BioTek	Winooski, VT, USA	-
ZetaView PMX 110	Particle Metrix	Mebane, NC, USA	-
Lionheart FX automated microscope	BioTek	Winooski, VT, USA	-
Luna-II automated cell counter	Logos Biosystems	Annandale, VA, USA	-
Odyssey infrared imaging system (LI-COR)	LI-COR	Lincoln, NE, USA	-

**Table 3:** The effect of BEV from uninfected and untreated RMs on U937 cell viability.

BEV Group	Concentration (µg)	Cell Viability (%)	*p* Value
VEH/SIV	20	91.2	0.0133
THC/SIV	20	91.6	0.0186
VEH/SIV	40	78.7	<0.0001
THC/SIV	40	81.2	0.0002
VEH/SIV	100	79.7	0.0005
THC/SIV	100	81.0	0.0010

## Supplementary Materials

The following are available online at https://www.mdpi.com/2073-4409/9/10/2243/s1, Figure S1: UV–vis absorbance profile for each individual RM for VEH/SIV group. Figure S2: UV–vis absorbance profile for each individual RM for THC/SIV group. Figure S3: UV–vis absorbance profile for each individual RM for THC only group. Figure S4: Raw NTA data analysis of BEVs. Figure S5: Raw NTA analysis of each individual RM for the VEH/SIV group. Figure S6: Raw NTA analysis of each individual RM for the THC/SIV group. Figure S7: Raw NTA analysis of each individual RM for the THC only group. Figure S8: Tetraspanin WB of each individual RM for the VEH/SIV and THC/SIV groups.
