# Supplementary material for: Long-Term Low-Dose Delta-9-Tetrahydrocannbinol (THC) Administration to Simian Immunodeficiency Virus (SIV) Infected Rhesus Macaques Stimulates the Release of Bioactive Blood Extracellular Vesicles (EVs) that Induce Divergent Structural Adaptations and Signaling Cues
Source: Cells. 2020 Oct 6;9(10):2243. doi: 10.3390/cells9102243 (PMC7599525; doi:10.3390/cells9102243)
Supplement: Supplementary file 1 [file cells-09-02243-s001.pdf]

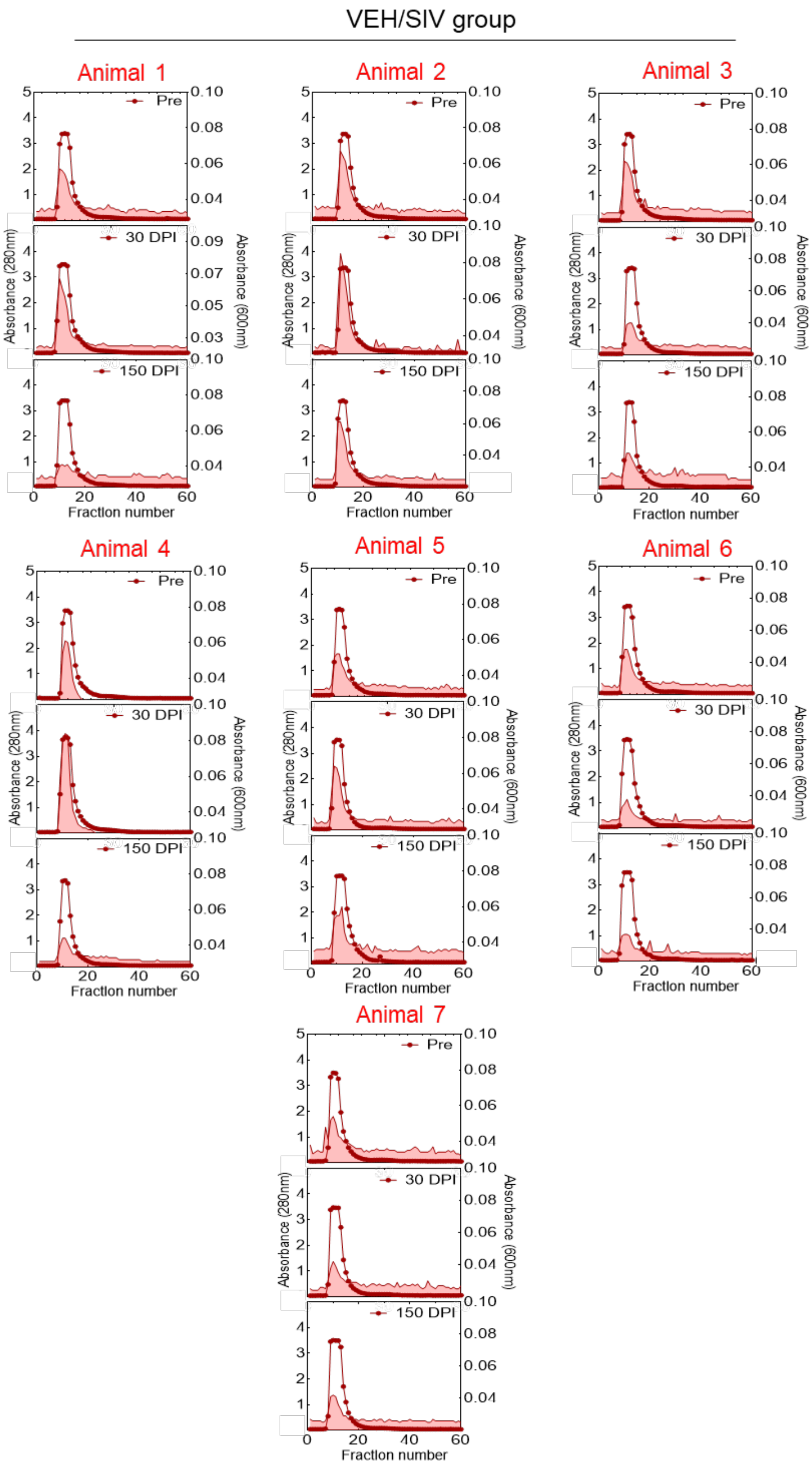

Figure S1: UV-Vis absorbance profile for individual VEH/SIV group.

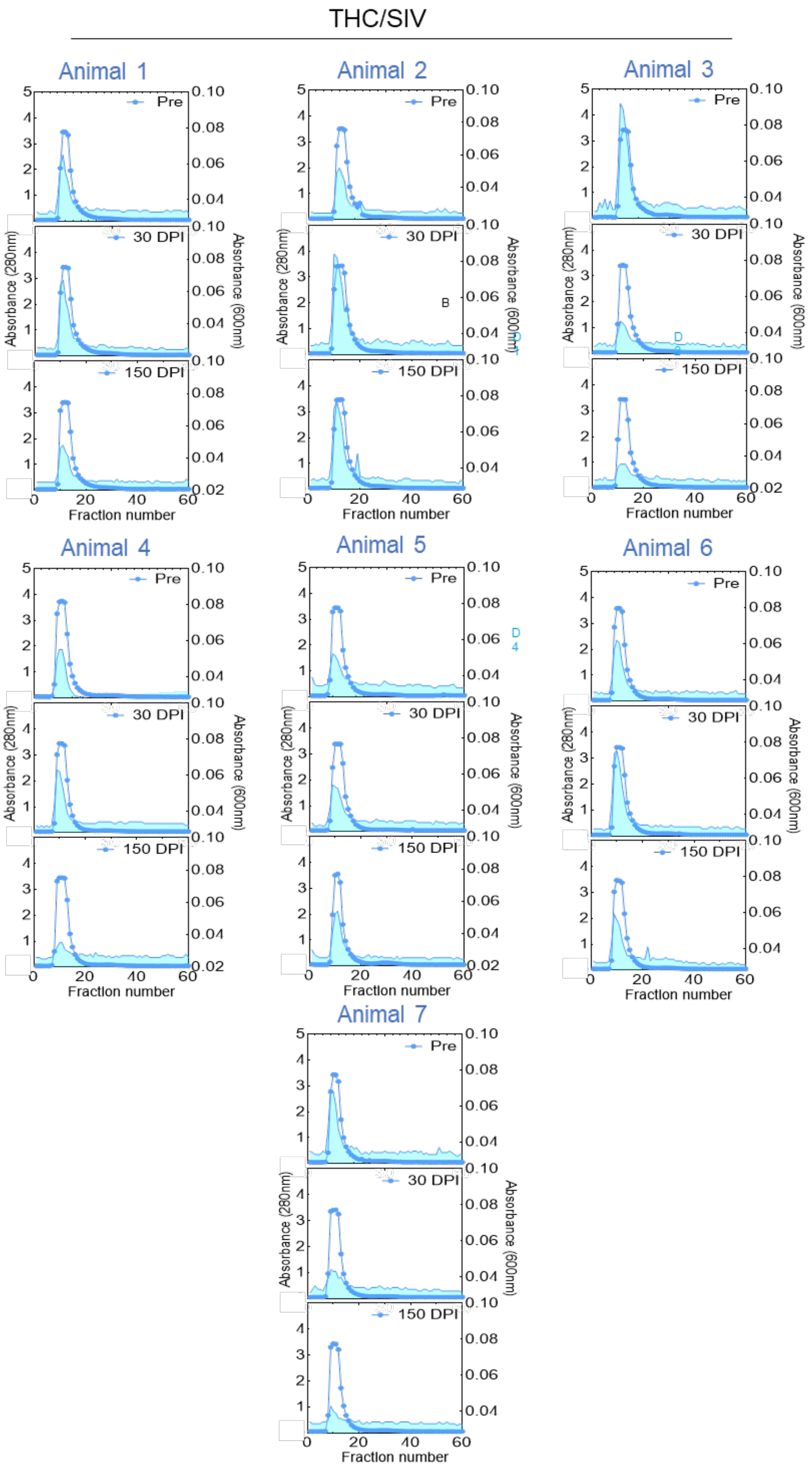

Figure S2: UV-Vis absorbance profile for individual THC/SIV group.

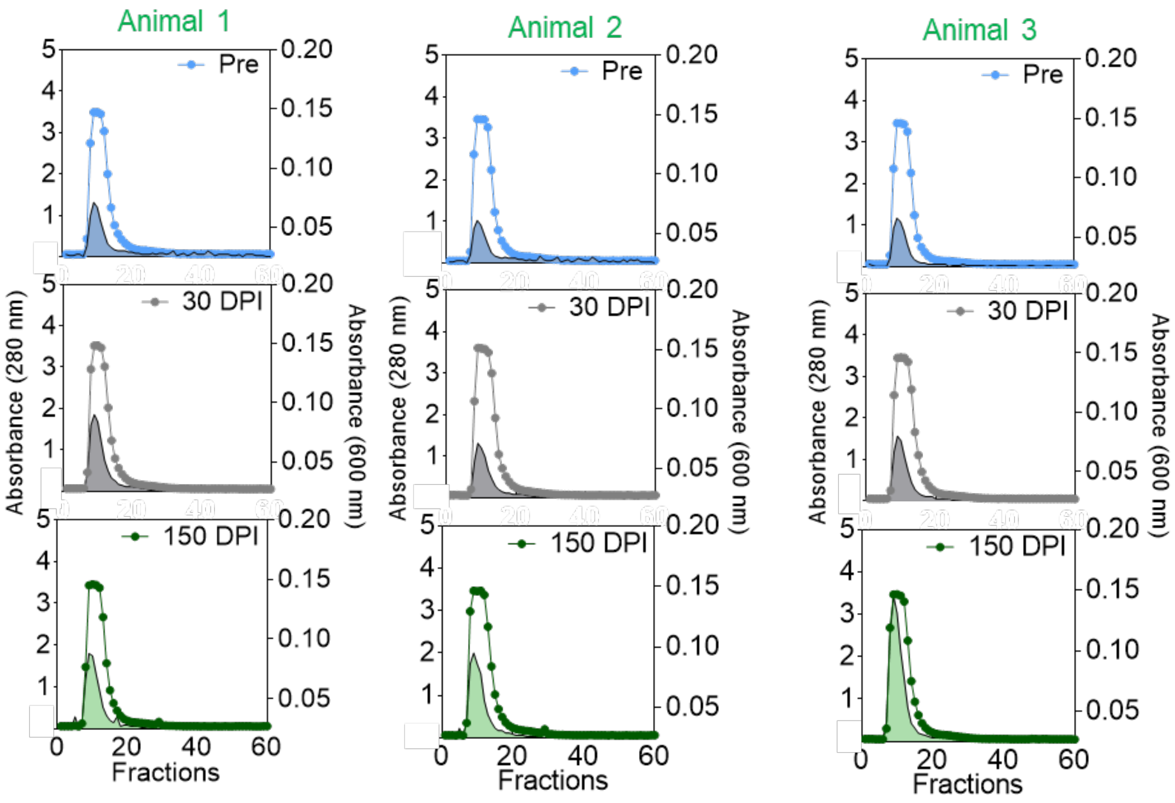

Figure S3: UV-Vis absorbance profile for individual THC only group.

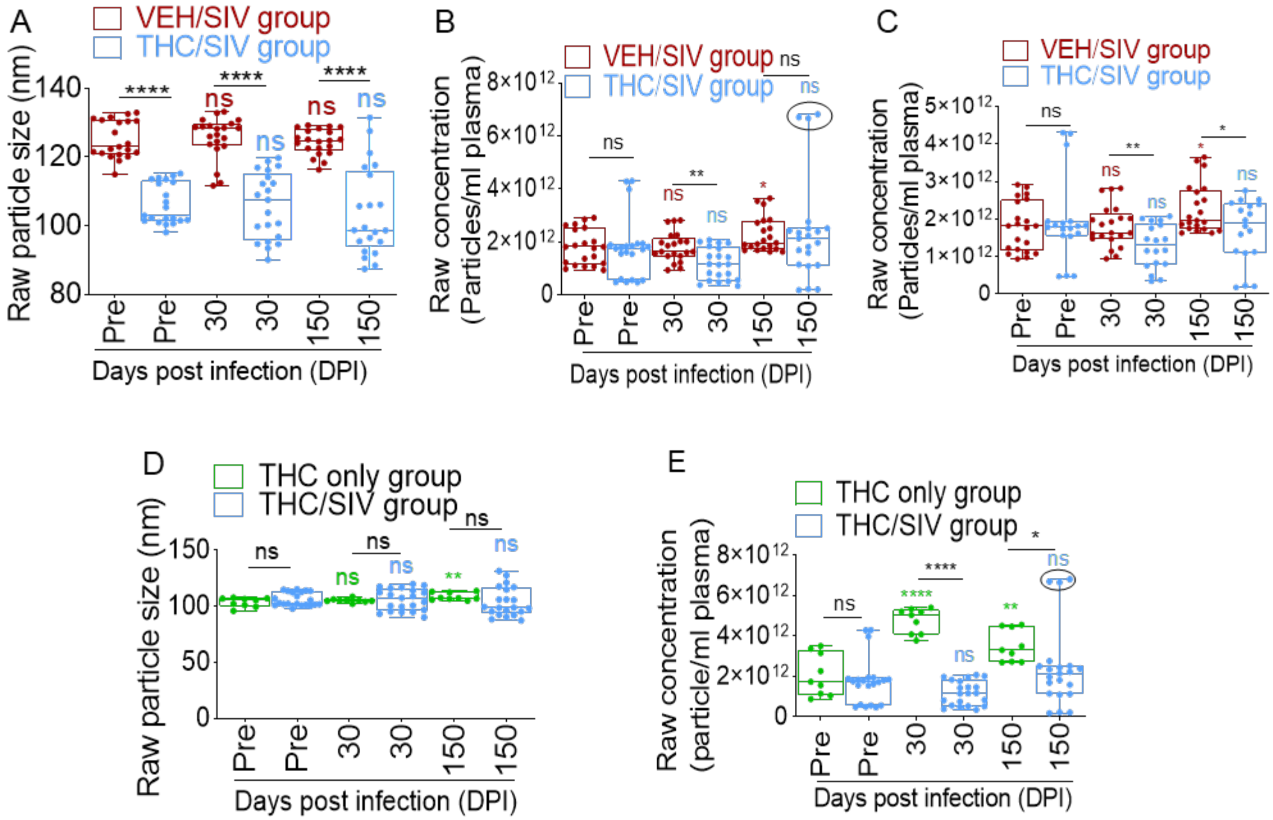

Figure S4: Raw NTA data analysis of BEVs: (A) Size and (B) concentration comparison between SIV group and SIV-THC group. (C) Concentration comparison without the outlier RM. (D) Size and (E) concentration comparison between THC group and THC/SIV group. Ordinary one-way ANOVA (Brown-Forsythe and Bartlett's tests, with Sidak's multiple comparisons test) was used to determine the statistical significance within the group. Binary Student's t-tests (Welch's correction) was used between groups for each of the time points in each group. **** $p<0.001$, *** $p<0.005$, ** $p<0.01$, * $p<0.05$, ns non-significant.

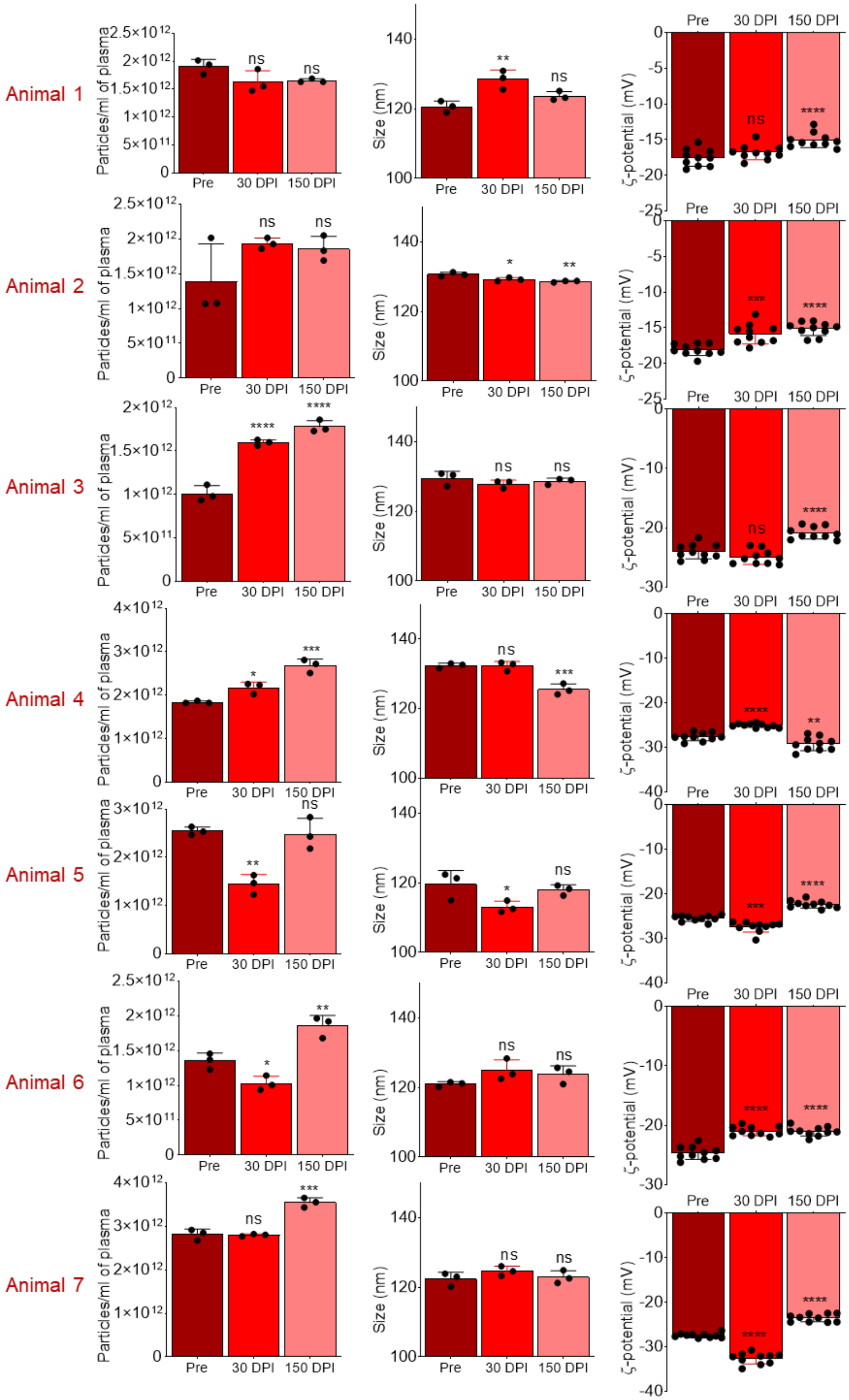

Figure S5: Raw NTA analysis of each individual RM for VEH/SIV group.

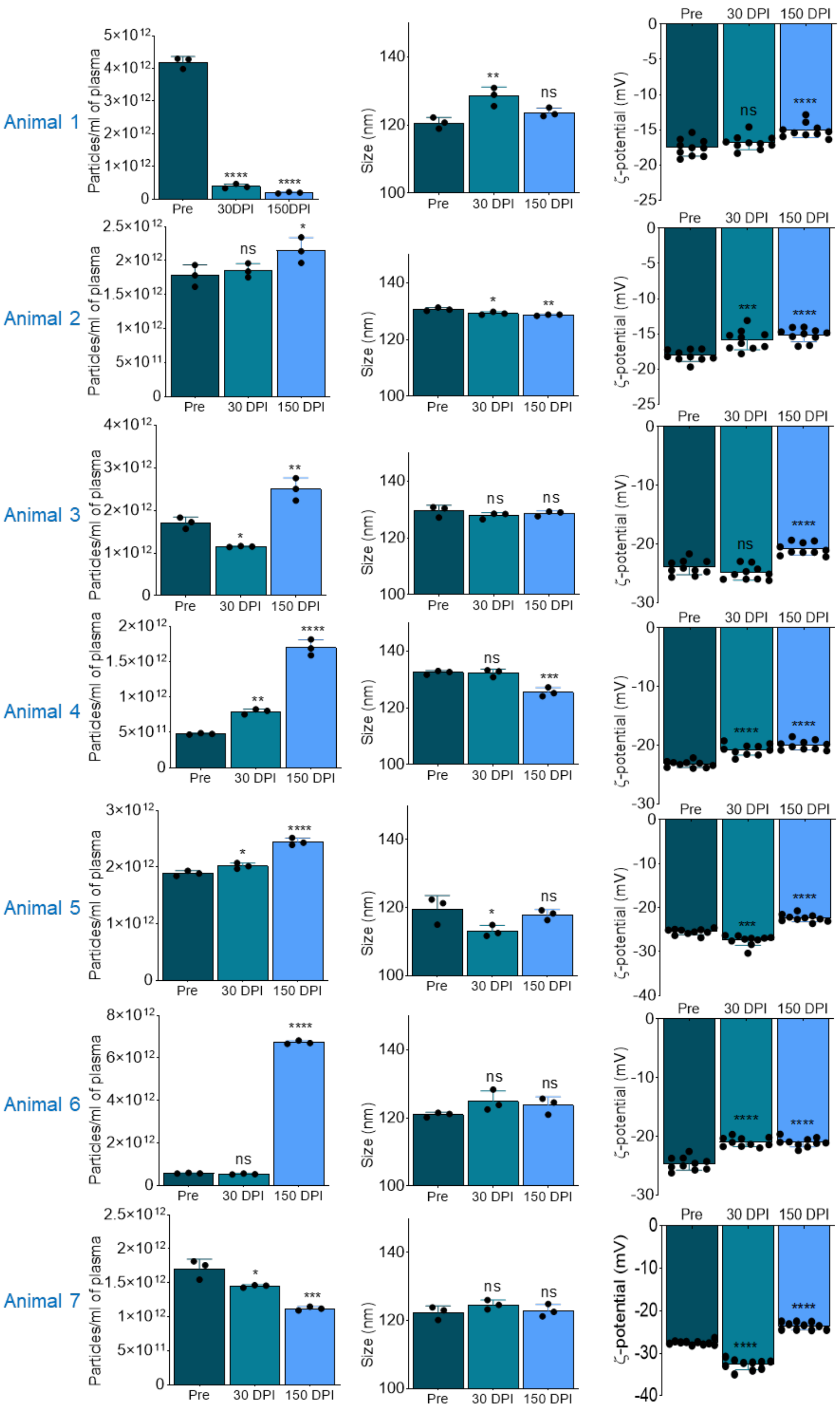

Figure S6: Raw NTA analysis of each individual RM for THC/SIV group.

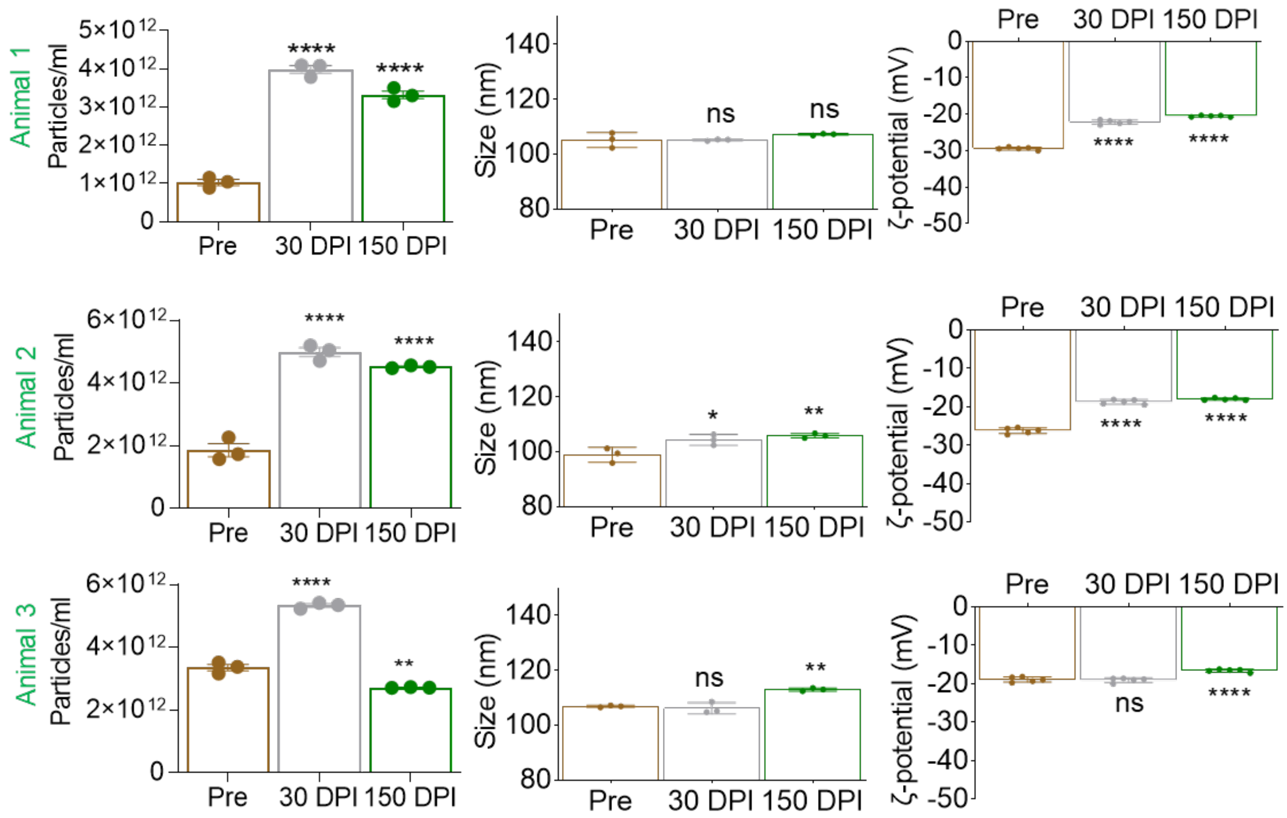

Figure S7: Raw NTA analysis of each individual RM for THC only group.

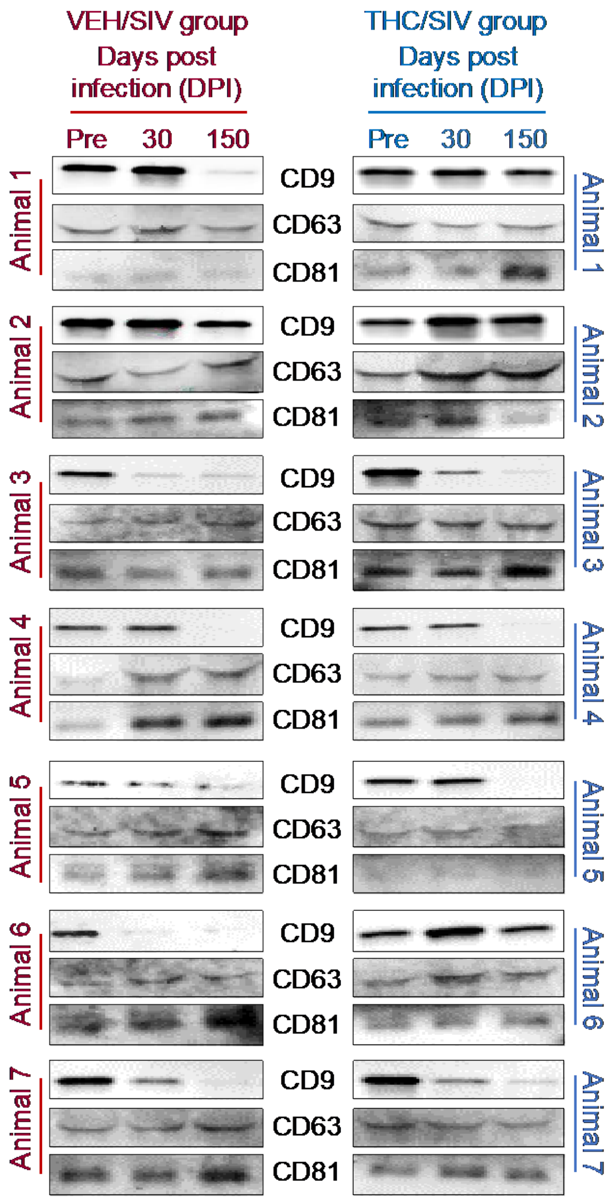

Figure S8. Tetraspanin WB of each individual RM for VEH/SIV and THC/SIV group.
